# Analysis of goalkeepers’ game performances at the 2016 European Football Championships

**DOI:** 10.17159/2078-516X/2020/v32i1a8283

**Published:** 2020-01-01

**Authors:** A Kubayi

**Affiliations:** Department of Sport, Rehabilitation and Dental Sciences, Faculty of Science, Tshwane University of Technology, Pretoria, South Africa

**Keywords:** physical performance, distance, technical skills, matches

## Abstract

**Background:**

Despite a substantial body of literature on the physical and technical demands of outfield players in football, there is little information regarding the performance of goalkeepers.

**Objective:**

The aim of this study was to analyse the game performance profiles of goalkeepers at the 2016 European Football Championships.

**Methods:**

A total of 30 goalkeepers from 15 games played during the 2016 European Football Championships were analysed using the InStat® video tracking system.

**Results:**

The results showed that goalkeepers covered a mean total distance of 4819 m, ranging from 4036 m to 6640 m. Overall, 68% of distance travelled was attributed to walking, whereas 0.8% was due to high-intensity activities. The goalkeepers of teams that lost matches covered significantly *(p* < 0.05) longer distances while sprinting than those of teams that drew or won the matches. Goalkeepers of teams that drew significantly (*p* < 0.05) had a greater number of passes than those goalkeepers of teams that won or lost.

**Conclusion:**

The current results have implications for soccer coaches to structure training sessions and tactical strategies for goalkeepers. The ability of goalkeepers to meet the physical and technical demands of a match could directly influence the successful execution of skills and the outcome of the competition.

The primary function of a soccer goalkeeper is to defend his/her goal, while the secondary role is to initiate attack through ball distribution.^[1,2]^ One of the greatest challenges a goalkeeper faces is that one mistake can cost the team its success. Almost every situation in which the goalkeeper is called into play is a high-pressure event, as it may potentially be a losing or winning situation in the match.^[1]^ Thus, a goalkeeper needs to possess a unique physical and technical profile, and it is likely that further details about their match-play and training demands would benefit practitioners seeking to optimise a training prescription for this playing population.^[2]^

Over the last decade, technological developments have seen the implementation of increasingly advanced video and motion analysis systems for collecting data on the physical and technical demands of soccer players.^[3]^ A number of studies has predominately focused on investigating the running demands of outfield players,^[2–4]^ with players covering total distances of 8−12 km during a soccer game.^[5]^ Conversely, limited research has analysed the physical demands of soccer goalkeepers. A review by White et al.^[2]^ demonstrated that goalkeepers cover total distances of four to six km during a match and appear not to experience between-half reductions in physical performance as the match progresses.

In addition to the reduced distances covered by goalkeepers, research has also found that the majority of this distance is covered at low-intensity levels, such as walking or jogging.^[6]^ With respect to comparisons between players, it has been found that goalkeepers performing at an international tournament spend approximately 98% of the game in the low-intensity threshold, compared to outfield players who spend approximately 83% of their time in this intensity zone.^[7]^ Furthermore, goalkeepers in the English Premier League were found to only spend 1% of their time in high-speed running (i.e. between 19.9 and 25.2 km/h), which was accounted for by approximately 10 high-speed runs and two sprints of less than 10 metres (>25.2 km/h) per match.^[6]^ While goalkeepers may only perform two short sprints per match, these actions could represent important phases of play directly related to key situations that can influence the score and outcome of the match.^[2]^

Despite there being few studies on the physical demands of goalkeepers in soccer matches,^2^,^6^ there is little information on the technical performance of goalkeepers during match-play. It is essential to consider the technical aspects of the goalkeepers ^[4,6]^ because this information can better predict successful team performance in soccer compared to purely physical parameters.^[8]^ Therefore, research on both physical and technical performances of goalkeepers could assist soccer coaches to better understand their specific match-related activities and training programmes can be modified appropriately.^[3,4]^ The purpose of this investigation was to analyse the game performance profiles of goalkeepers at the European Football Championships.

## Methods

### Match sample and data collection

A total of 30 goalkeepers from 15 games played during the 2016 European Football Championships were analysed using the InStat® video tracking system (https://instatsport.com/football). Match activities were recorded using two video cameras installed on tripods set at the end of each stand. Video recordings from each camera fully covered separate halves of the field. The reliability of the InStat® tracking system has been demonstrated in previous research.^[9]^ Ethical clearance was received from the author’s institutional ethics committee.

### Physical and technical indicators

Physical indicators of goalkeepers were categorised as follows: (a) walking (0–7 km/h), (b) jogging (7.1–14.5 km/h), (c) running (14.6–20 km/h), (d) high-speed running (20.1–25 km/h) and (e) sprinting (>25 km/h). High-intensity activity was defined as high-speed running and sprinting (>20.1 km/h). Distances in attack and defence were calculated according to whether the goalkeeper was in possession of the ball or not. All distances in various categories were combined to calculate the total distance covered.

The technical indicators included variables such as save (the goalkeeper prevents the ball from entering the goal with any part of his body), pass (an intentional played ball from the goalkeeper to his teammate, including ball throwing from the hand), pass accuracy (%) (a ratio calculated from successful passes divided by all passes), aerial duels won (%) (two players competing for a ball in the air – for it to be an aerial duel, both players must jump and challenge each other in the air and have both feet off the ground), tackle (act of gaining possession from an opposition player who is in possession of the ball), lost ball (the goalkeeper lost ball possession due to a mistake/poor control, including turnovers, dispossession, and unsuccessful passes), ball recovery (the event given at the start of a goalkeeper’s recovery of ball possession from opponents from open play), foul drawn (where the goalkeeper is fouled by an opponent), and yellow-card (where the goalkeeper is booked by the referee due to illegal actions).^[4,10]^

### Statistical analysis

Data were reported as means ± standard deviations. One-way analysis of variance was used to examine differences on the physical and technical parameters of goalkeepers based on match outcomes (i.e. win, lose or draw). In addition, if the *F*-ratio was significant at *p*≤0.05, then Tukey’s HSD post hoc analysis was conducted. The effect size (ES) was used to determine the magnitudes of the studied variables. ES was grouped as follows: trivial (<0.20), small (0.20–0.59), moderate (0.60–1.19), large (1.20–2.00), and very large (>2.00). ^[11]^ All analyses were computed using the IBM Statistical Package for the Social Sciences (SPSS), version 25.0.

## Results

Table 1 presents descriptive characteristics of distances covered at different intensities by goalkeepers. The mean total distance covered by goalkeepers was 4819 m (range = 4036 m to 6640 m). Walking accounted for 68% of distance travelled during matches, with only 0.8% of distance covered spent in high-intensity activities, such as high-speed running and sprinting. There were minimal differences in the distances covered during the first half (2412 ± 281 m) and second half (2408 ± 323 m) of matches, with trivial effect (ES = 0.01). Goalkeepers covered longer distances during attacking phases of play (1660 ± 463 m), compared with defending (1569 ± 436 m) phases (ES = 0.20, small effect).

Table 2 shows distances covered by goalkeepers at different intensities, as well as technical indicators based on match outcome. Goalkeepers of teams that lost covered significantly greater distances while sprinting (6.0 ± 9.9 m) compared with those of teams that drew or won games (*F* [2, 27] = 3.48, *p* = 0.05). Post hoc comparisons showed that the mean score for a sprinting distance of goalkeepers of teams that lost was significantly higher than for teams that won or drew. Goalkeepers of teams that drew covered greater distances associated with walking (3333 ± 380 m) and high-speed running (49 ± 31 m). Goalkeepers of the teams that won covered longer distances when defending (1480 ± 422 m), while those of teams that lost (1591 ± 529 m) or drew (1953 ± 466 m) covered greater distances associated with attacking phases.

With regard to the technical variables, a statistically significant difference was observed on passes (*F* [2, 27] = 4.61, *p* = .01). The post hoc comparisons showed that the mean score of the passes for the goalkeepers of teams that drew (40 ± 9) was significantly different from those of teams that won (29 ± 8, ES = 1.21, large effect) or lost (28 ± 8, ES = 1.45, large effect). Goalkeepers of teams that won had a higher number of aerial duels won, albeit not significant, than those of teams that lost (ES = 1.71, large effect).

## Discussion

The aim of this study was to analyse the game performance profiles of goalkeepers at the 2016 European Football Championships. Regarding physical parameters, goalkeepers covered an average total distance of 4819 m during a match. This result is lower than that of previous findings, which reported that goalkeepers covered a total distance of 5611 m during match-play.^[6]^ The current study demonstrated that there are minimal differences between the halves of the game in terms of the distance covered by goalkeepers. This observation is consistent with that of Di Salvo et al.^[6]^ who found that the physical performance for goalkeepers is mirrored across the two halves. Regardless of variations between studies, these distances represent about 50% of those covered by outfield players and may explain why no between-half declines in total distance have been observed within any intensity threshold for international goalkeepers.^[2]^

As indicated in previous studies,^[2,6]^ goalkeepers walked for 68% of the total match duration and spent only 0.8% of the match in high-intensity activities. This finding suggests that as goalkeepers work in a limited space (i.e. in the defensive penalty area), it may be difficult for them to display a large number of high-intensity actions during a match.^[6]^ Despite their low high-intensity running distances, this may, however, represent actions which can have a direct influence on the outcome of a match,^[2]^ and therefore coaches should design training sessions involving high-intensity, game-specific activities. Regarding running performance and match outcome, the finding that the goalkeepers of the teams that lost covered significantly greater distances in sprinting compared to the goalkeepers of the teams which won or drew, could be explained by the fact that they may push forward when losing, thus reaching their maximal physical capacity in the hope of potentially drawing or winning the game.^[12]^

The present data further showed that goalkeepers of teams that drew covered a greater distance in the defending phases of play than those of the teams that lost or won. From a defensive perspective, this finding could be associated to the fact that teams that draw may adopt a more defensive strategy to ensure that they do not concede and potentially lose the game. In this context, players may adopt a ball retention strategy,^[13]^ which could result in higher distance covered when defending during a match. Maintaining possession of the ball is one of the most physically demanding playing styles because, if executed appropriately, players (including goalkeepers) may have to expend more energy during a match.^[14]^

Furthermore, the goalkeepers of teams that drew or won had a greater number of passes than those of teams that lost. In modern soccer, goalkeepers need to be proficient in their ball control skills, such as passing, so that without the option to use their hands, back-passes from teammates are secured to better deal with opponent pressure.^[15]^ Goalkeepers of teams that won had a higher number of successful aerial duels, with a large magnitude, compared to the goalkeepers of teams that lost or drew. Liu et al.^[10]^ reported that teams which are effective in dealing with aerial duels are more likely to dominate both the attacking and defending phases, eventually leading to a match win.

## Conclusion

This study suggests that goalkeepers perform most match activities at a low intensity, with only a small proportion of actions executed at high-intensity. Goalkeepers of teams that lost covered significantly greater distances while sprinting compared to those of the teams that drew or won. The goalkeepers of teams that won had a higher number of ball recoveries than those of teams that lost and won. Therefore, the results of this study are important for soccer coaches in designing training programmes for goalkeepers so that they can meet the physical and technical demands of a game which could directly influence the competition’s outcome.

## Figures and Tables

**Table 1:** The mean distances (m) covered by goalkeepers during a match relative to the different intensities, game period and phases of play (n=30)

	Mean	Standard Deviation	Minimum	Maximum
**Total distance (m)**	4819	580	4036	6640
Walking	3277	367	2590	3990
Jogging	1272	395	744	2127
Running	230	108	81	569
High-speed running	39	28	11	113
Sprinting	2.2	6.5	0	30
First half	2412	281	2048	3080
Second half	2408	323	1971	3560
In defence	1569	436	881	2645
In attack	1660	463	874	2767

m, metres

**Table 2:** Physical and technical indicators of goalkeepers according to the match outcome (n=30)

	Win	Lose	Draw	Significance (p-value)
**Physical indicators**				
Total distance (m)	4808 ± 433	4787 ± 771	4880 ± 522	0.94
Walking (m)	3247 ± 448	3267 ± 292	3333 ± 380	0.88
Jogging (m)	1304 ± 285	1241 ± 448	1272 ± 491	0.94
Running (m)	231 ± 83	231 ± 135	226 ± 112	0.99
High-speed running (m)	27 ± 16	43 ± 32	49 ± 31	0.20
Sprinting (m)	0.0 ± 0.0	6.0 ± 9.9#	0.0 ± 0.0	0.04*
In defence (m)	1480 ± 423	1432 ± 354	1880 ± 447	0.06
In attack (m)	1518 ± 305	1591 ± 529	1953 ± 466	0.10
**Technical indicators**				
Save (n)	4.6 ± 2.5	3.8 ± 1.7	2.9 ± 2.0	0.21
Pass (n)	29 ± 9	28 ± 8	40 ± 9#	0.01*
Pass accuracy (%)	87 ± 4	88 ± 9	89 ± 6	0.83
Aerial duel won (%)	64 ± 51	18 ± 41	13 ± 35	0.21
Tackle (n)	0.09 ± 0.30	0.18 ± 0.40	0 ± 0	0.45
Lost ball (n)	3.3 ± 1.4	2.9 ± 2.8	3.3 ± 2.0	0.90
Ball recovery (n)	5.6 ± 2.0	5.5 ± 3.0	6.6 ± 4.0	0.68
Foul drawn (n)	0.09 ± 0.30	0.18 ± 0.40	0.25 ± 0.46	0.68
Yellow card (n)	0.09 ± 0.30	0 ± 0	0 ± 0	0.44

Data are expressed as mean ± standard deviation.

* Significant at p<0.05;

# Significantly higher than other teams at p<0.05. m, metres; %, percentage; n, number
